# Insight into the substrate specificity change caused by the Y227H mutation of α-glucosidase III from the European honeybee (*Apis mellifera*) through molecular dynamics simulations

**DOI:** 10.1371/journal.pone.0198484

**Published:** 2018-06-04

**Authors:** Pratchaya Pramoj Na Ayutthaya, Chanpen Chanchao, Surasak Chunsrivirot

**Affiliations:** 1 Department of Biology, Faculty of Science, Chulalongkorn University, Pathumwan, Bangkok, Thailand; 2 Structural and Computational Biology Research Group, Department of Biochemistry, Faculty of Science, Chulalongkorn University, Pathumwan, Bangkok, Thailand; 3 Department of Biochemistry, Faculty of Science, Chulalongkorn University, Pathumwan, Bangkok, Thailand; Universidade Nova de Lisboa Instituto de Tecnologia Quimica e Biologica, PORTUGAL

## Abstract

Honey from the European honeybee, *Apis mellifera*, is produced by α-glucosidases (HBGases) and is widely used in food, pharmaceutical, and cosmetic industries. Categorized by their substrate specificities, HBGases have three isoforms: HBGase I, II and III. Previous experimental investigations showed that wild-type HBGase III from *Apis mellifera* (WT) preferred sucrose to maltose as a substrate, while the Y227H mutant (MT) preferred maltose to sucrose. This mutant can potentially be used for malt hydrolysis because it can efficiently hydrolyze maltose. In this work, to elucidate important factors contributing to substrate specificity of this enzyme and gain insight into how the Y227H mutation causes substrate specificity change, WT and MT homology models were constructed, and sucrose/maltose was docked into active sites of the WT and MT. AMBER14 was employed to perform three independent molecular dynamics runs for these four complexes. Based on the relative binding free energies calculated by the MM-GBSA method, sucrose is better than maltose for WT binding, while maltose is better than sucrose for MT binding. These rankings support the experimentally observed substrate specificity that WT preferred sucrose to maltose as a substrate, while MT preferred maltose to sucrose, suggesting the importance of binding affinity for substrate specificity. We also found that the Y227H mutation caused changes in the proximities between the atoms necessary for sucrose/maltose hydrolysis that may affect enzyme efficiency in the hydrolysis of sucrose/maltose. Moreover, the per-residue binding free energy decomposition results show that Y227/H227 may be a key residue for preference binding of sucrose/maltose in the WT/MT active site. Our study provides important and novel insight into the binding of sucrose/maltose in the active site of *Apis mellifera* HBGase III and into how the Y227H mutation leads to the substrate specificity change at the molecular level. This knowledge could be beneficial in the design of this enzyme for increased production of desired products.

## Introduction

**Fig 1:**
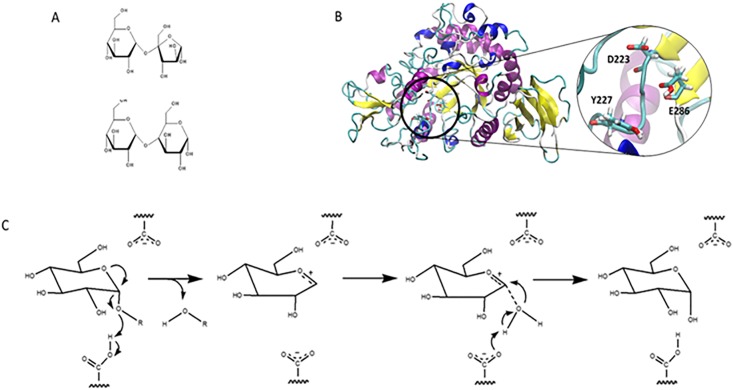
(A) Structures of maltose (bottom) and sucrose (top). (B) Homology model of *Apis mellifera* HBGase III. Catalytic residues are shown in licorice with name coloring. (C) Proposed hydrolysis reaction of α-glucosidase linkage via an oxocarbenium ion intermediate.

*Apis mellifera* produces honeybee α-glucosidase (HBGase), which is an exo-type carbohydrase that catalyzes the cleavage of an α-glycosidic linkage of polysaccharides from the non-reducing end and produces α-glucose as a storage product in honey [1]. Categorized by their substrate specificities and locations in internal organs, HBGase possesses three isoforms: HBGase I, II, and III, located in ventriculus, hemolymph, and hypopharyngeal gland, respectively [2,3]. α-Glucosidases (E.C. 3.2.1.20, α-D-glucoside glucohydrolase) can also be classified into two groups based on substrate specificities and locations of conserved regions on the primary structure [4]. The first group is found in brewer’s yeast (*Saccharomyces cerevisiae*) and bacterial enzymes [5,6] and prefers hydrolyzing a heterogeneous substrate (such as sucrose) to maltooligosaccharide and can hydrolyze α-glucans with low activity. The second group is found in animal and mold enzymes [7]; these α-glucosidases prefer the hydrolysis of maltooligosaccharide to that of a heterogeneous substrate and can also hydrolyze α-glucans [6]. HBGase III belongs to the first group because it prefers sucrose to maltose as a substrate (Fig 1A) [8]. HBGase III is a member of glycoside hydrolase families 13, and its catalytic residues were proposed to be D223 and E286 (Fig 1B) [8]. HBGase III can potentially be used in the production of isomaltooligosaccharides (IMOs) or “industrial” IMO via transglycosylation of hydrolyzed starch [9]. Since humans lack the enzymes that can digest IMOs, these products are included in many commercial food supplies including protein/fiber bars, shakes, and other dietary supplements that are appropriate for diabetic patients and low-carbohydrate consumers [9].

The oxocarbenium ion mechanism has been proposed for the catalytic reaction mechanism of many carbohydrate-degrading enzymes such as glucosidases, lysozymes, and amylases [10]. Fig 1C shows the proposed oxocarbenium ion intermediate mechanism where the carboxyl and carboxylate groups cooperatively participate in the hydrolytic reaction. The carboxylate group is proposed to promote the formation of the oxocarbenium ion and to stabilize the intermediate. The carboxyl group donates its proton to the oxygen atom of the glycosidic linkage to promote the release of a hydrolytic product and the formation of an oxocarbenium ion [10]. This mechanism is strongly supported by the α-secondary kinetic isotope effects observed in the hydrolytic reactions of lysozyme, glucoamylase, and α-glucosidases [10]. Moreover, this mechanism can be used to explain the reaction mechanisms of both the “retaining” and the “inverting” enzymes [10].

With regard to substrate specificity, Ngiwsara *et al*. found that HBGase III from *Apis mellifera* (WT) preferred sucrose to maltose as a substrate, while its Y227H mutation caused a drastic change in the substrate specificity with the Y227H mutant (MT) preferring maltose to sucrose as a substrate at pH 5.5 and 310 K [8]. For WT, the initial reaction velocity of maltose was approximately 24% of that of sucrose, indicating that WT preferred sucrose to maltose as a substrate. For MT, the initial reaction velocity of sucrose decreased to approximately 57% of that of WT, while that of maltose significantly increased and was higher than that of sucrose, indicating that MT preferred maltose to sucrose as a substrate. Therefore, Y227 and H227 were proposed to be involved in the substrate preferences for sucrose and maltose, respectively [8]. Moreover, this mutant can potentially be used for malt hydrolysis because it can efficiently hydrolyze maltose [11]. However, the molecular level understanding of the mechanism through which the Y227H mutation changes the substrate specificity of HBGase III is lacking.

In this study, the homology model of *Apis mellifera* HBGase III was constructed, and three independent molecular dynamics simulations runs were performed for sucrose/WT, maltose/WT, sucrose/MT, and maltose/MT complexes at experimental pH and temperature [8] in order to investigate their binding dynamics and free energies as well as to gain insight on how the Y227H mutation changes the substrate specificity of *Apis mellifera* HBGase III. This knowledge will be beneficial for the design of HBGase III with improved activity for desired products.

## Materials and methods

### Structural preparation

The SWISS-MODEL server [12] was employed to construct the homology model of *Apis mellifera* HBGase III (residue P23 to D561), using the structure of isomaltulose synthase from *Erwinia rhapontici* (PDB: 4HPH) as a template [13]. The N-terminus and C-terminus were capped with ACE and NME groups, respectively. Ramachandran plots created by the RAMPAGE server [14] were used to assess the quality of the constructed homology model. A majority of the residues were found in the favored region (92.20%) and allowed region (4.30%), indicating that the constructed homology model was reasonable (S1 Fig). Moreover, the catalytic residues (D223 and E286) of this homology model were found to be in the positions that are appropriate for hydrolysis. To create the Y227H mutant of *Apis mellifera* HBGase III, the SWISS-PDB viewer program was used [15]. Using the H++ server [16], structures of the wild type and the Y227H mutant were protonated at the experimental pH of 5.5. Their atom types and force field parameters were assigned based on the AMBER ff14SB force field, and their hydrogen and missing atoms were added using the LEaP module in AMBER14 [17]. The structures of maltose and sucrose were obtained from the structures with the PDB IDs of 3WY4 [18] and 4HPH [13], respectively. The GLYCAM06j-1 force field was used to assign the atom types and force field parameters [19]. To determine the binding conformations of maltose and sucrose in the active sites of WT and MT, Autodock vina [20] was employed with the grid box of 20x20x20 Å^3^ and 1 Å spacing. To determine whether the Autodock vina and its parameters were appropriate for our systems, the crystal maltose and sucrose were redocked into the active sites of α-glucosidase (3WY4) from *Halomonas sp*. H11 [18] and isomaltulose synthase (4HPH) from *Erwinia rhapontici* [13], respectively. The best docked and crystal binding conformations were compared and found to be reasonably similar, with RMSD values of 0.42 Å and 0.43 Å for maltose and sucrose, respectively (S2 Fig). Therefore, Autodock vina and its corresponding parameters were employed to determine the binding conformations of the four complexes. For each complex, the docked conformation with the best affinity was selected (S3 Fig and S1 Table), and the LEaP module in AMBER14 was used to prepare the structure for molecular dynamics (MD) simulations.

### Molecular dynamics simulations

The AMBER14 package was employed for structural minimizations and MD simulations. Each complex was placed in an isomeric truncated octahedron box of TIP3P water with the buffer distance of 13 Å and neutralized by additional Na^+^ cations. Each system was minimized using the five-step procedure described below. Employing different restraints on the protein structure, all simulation steps consisted of 1,000 steepest-descent minimization cycles and 1,000 conjugate-gradient minimization cycles. Initially, harmonic restraints with the force constant of 10 kcal/(mol Å^2^) were used to immobilize the protein structure, excluding hydrogen atoms, while solvent molecules were allowed to relieve unfavorable contacts with other molecules. Then, the backbone of the protein was immobilized using harmonic restraints with the force constants of 10, 5, and 1 kcal/(mol Å^2^). Finally, the energy of the whole system was minimized without any positional restraints. In the NVT ensemble, each system was heated from 0 to 310 K (experimental temperature) during a 200 ps MD simulation using weak harmonic restraints with the force constant of 10 kcal/(mol Å^2^) on the protein backbone. With no restraints, each system was further equilibrated for 300 ps at 310 K in the NVT ensemble. In the NPT ensemble, each system was simulated for 85 ns. Langevin dynamics with the collision frequency of 1 ps^-1^ was used to control the temperature in all simulations. The pressure in the NPT simulations was maintained at the average pressure of 1 atm by an isotropic position scaling algorithm with the relaxation time of 2 ps. A cutoff of 12 Å was applied for non-bonded interactions, and the particle mesh Ewald method with the default parameters was used to compute the long-range electrostatic interactions. For all simulations, the random number generator was reseeded [21]. To remove the bond-stretching freedom for all bonds involving hydrogen, the SHAKE algorithm [22] with the tolerance parameter of 10^−5^ Å was employed, thereby allowing the use of a 0.002 ps time step. Three independent simulations were carried out using different seeding numbers.

To measure the system stabilities during the MD simulations, the root-mean-square deviations (RMSDs) were computed for all systems. The binding residues in the WT and MT binding sites were defined to be the residues within 5 Å of maltose/sucrose in maltose-WT, maltose-MT, sucrose-WT, and sucrose-MT complexes. Therefore, the binding residues consisting of residues 81, 82, 84, 121, 124, 167, 168, 187, 191, 221, 223, 224, 227, 252, 254, 286, 308, 312, 347, 348, 399, and 417 were used for binding analyses in all four systems. CPPTRAJ [23] was employed to calculate hydrogen bond occupations between maltose/sucrose and proteins. For hydrogen bond occupation analysis, a hydrogen bond was considered to occur if the following criteria were met: (i) a proton donor-acceptor distance ≤ 3.00 Å and (ii) a donor-H-acceptor bond angle ≥ 135°.

Molecular mechanics generalized-born surface area (MM-GBSA) method [24, 25] was used for per-residue binding free energy decomposition of the binding residues and the total binding free energies as well as their energy components for all systems. The MM-GBSA method is widely used to approximate the free energy of the binding of small ligands to macromolecules [26]. Previous studies showed that GBSA gave promising results in correctly ranking the molecules with known affinity to their target proteins [27–36] and distinguishing active molecules from inactive molecules [37, 38]. This method is also stable and reproducible [26]. Moreover, it has been successfully used for rigorous decomposition of free energy into the contributions from different groups of atoms or types of interaction in various studies [28, 39–42].

## Results and discussion

### System stabilities

**Fig 2:**
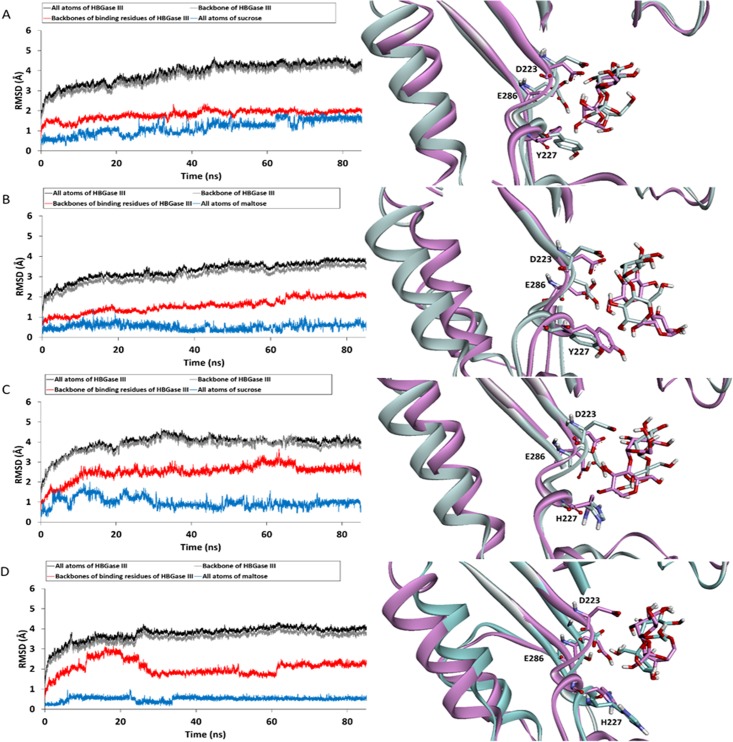
RMSD plots (left) and superimpositions between the representative structures, which are structures most similar to the average structures from the 65–85 ns trajectories, and the structures after minimization (right): (A) sucrose/WT complex, (B) maltose/WT complex, (C) sucrose/MT complex, and (D) maltose/MT complex.

Three independent molecular dynamics runs were performed on four systems: sucrose/WT, maltose/WT, sucrose/MT, and maltose/MT complexes. To determine the stabilities of these systems and identify appropriate trajectories for further analyses, the RMSD values of all atoms, backbone atoms of enzymes, backbone atoms of binding residues and all atoms of sucrose/maltose were calculated as shown in Fig 2, S4 and S5 Figs. The RMSD plots of all systems show that for all systems, the simulations were likely to reach equilibrium at approximately 65 ns. Therefore, the 65–85 ns trajectories were used for further analyses. The superimpositions between the representative structures of the complexes that are most similar to the average structures from the 65–85 ns trajectories and the initial structures after minimization are also shown in Fig 2. The positions and conformations of the initial and representative structures of sucrose in the WT active site appear to be more similar to each other than those of maltose in the WT active site, while those of maltose in the MT active site appear to be more similar to each other than those of sucrose in the MT active site. These initial results suggest that sucrose may not change its relative position and conformation in the WT active site as much as maltose, while maltose may not change its relative position and conformation in the MT active site as much as sucrose.

### Binding free energy calculations

To determine whether binding affinity is an important factor associated with substrate specificity of HBGase III, the average binding free energies (ΔG_bind_) and their components from the 65–85 ns trajectories from three independent runs were calculated (Table 1, S2 and S3 Tables). Based on the relative binding free energies, sucrose ranks better than maltose in WT binding, while maltose ranks better than sucrose in MT binding. These rankings support the experimental substrate specificity results that WT preferred sucrose to maltose as a substrate, while MT preferred maltose to sucrose [8]. These results suggest the importance of binding affinity for substrate specificity.

**Table 1:** Binding free energies and their components for sucrose/WT, maltose/WT, sucrose/MT, and maltose/MT complexes.

System	Binding free energy and its components (kcal/mol)							
	ΔE_vdw_	ΔE_ele_	ΔG_pol_	ΔG_np_	^a)^ΔG_solv_	-TΔS_tot_	^b)^ΔG_bind_	s.e.m. of ΔG_bind_
**Sucrose/WT**	-30.1	-114.9	110.9	-6.1	104.8	24.0	-16.2	1.2
**Maltose/WT**	-33.7	-99.4	103.3	-6.3	97.0	26.5	-9.6	1.7
**Sucrose/MT**	-25.7	-90.0	94.2	-5.1	89.1	22.5	-4.1	1.9
**Maltose/MT**	-29.7	-114	107.3	-6.0	101.4	24.0	-18.4	1.2

^a^ΔG_solv_ = ΔG_pol_ + ΔG_np_

^b^ΔG = ΔE_vdw_ + ΔE_ele_ + ΔG_solv_—TΔS_tot_

The main components contributing to the substrate binding affinities of all complexes are the electrostatic interactions (ΔE_ele_) because these terms have the most favorable values and their ranking is consistent with the calculated and experimental substrate specificities for all three runs. Other terms favoring sucrose/maltose binding are the van der Waals energy (ΔE_vdw_), which is in the ranges of -33.7 –-25.7, -34.8 –-27.9, and -29.7 –-23.8 kcal/mol for the first, second, and third runs, respectively, and the non-polar solvation term (ΔG_np_), which is in the ranges of -6.3 –-5.1, -6.4 –-5.1, and -6.1 –-4.7 kcal/mol for the first, second, and third run, respectively. The polar solvation term (ΔG_pol_) makes an unfavorable contribution to the ligand binding in the ranges of 94.2–110.9, 81.4–104.1, and 90.1–115.6 kcal/mol for the first, second, and third runs, respectively. The entropic contribution (-TΔS_tot_) is in the ranges of 22.5–26.5, 18.7–25.1, and 23.2–25.4 kcal/mol for the first, second, and third runs, respectively.

### Per-residue substrate-enzyme interactions

**Fig 3:**
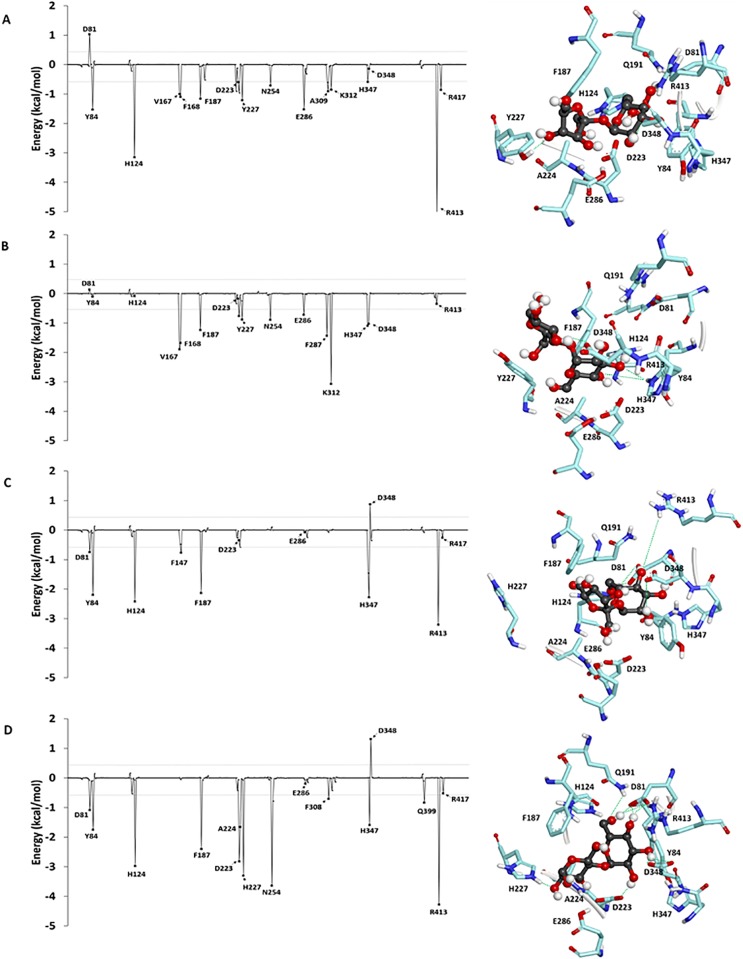
Per-residue decomposition of binding free energy contributions of (A) sucrose/WT complex, (B) maltose/WT complex, (C) sucrose/MT complex, and (D) maltose/MT complex.

To identify important binding residues that make major contributions to the calculated binding free energies, the values obtained by the decomposition of free energy on a per residue basis (${\Delta G}_{bind}^{residue}$) were computed as shown in Fig 3, S6 and S7 Figs. Overall, the values of ${\Delta G}_{bind}^{residue}$ of the binding residues of the sucrose/WT complex are more favorable than those of the maltose/WT complex, while those of the maltose/MT complex are more favorable than those of the sucrose/MT complex. These trends are consistent with the rankings of the relative binding free energies. The binding residues of the sucrose/WT complex contributing to favorable binding with ${\Delta G}_{bind}^{residue}$ less than -0.5 kcal/mol in all three independent runs are Y84, F187, D223, Y227, E286, D348, R413 and R417, while those of the maltose/WT complex are D81, Y84, F187, H347 and R413. Moreover, those of the sucrose/MT complex are Y84 and H347, while those of the maltose/MT complex are H124, F187, D223, H227, E286, F308, H347, R413 and R417. These results show that the number of favorable binding residues of the sucrose/WT complex is greater than that of the maltose/WT complex, while the number of favorable binding residues of the maltose/MT complex is greater than that of the sucrose/MT complex.

Furthermore, the value of ${\Delta G}_{bind}^{residue}$ of Y227 in the sucrose/WT complex is more favorable than that in the maltose/WT complex, while the ${\Delta G}_{bind}^{residue}$ value of H227 in the maltose/MT complex is more favorable than that of the sucrose/MT complex. For the sucrose/MT complex, H227 moved away from sucrose, suggesting that H227 may not bind well to sucrose in the MT active site (Fig 3C). These results support the previous study, which proposed that Y227 and H227 were involved in the substrate preference for sucrose and maltose, respectively [8]. Additionally, the values of ${\Delta G}_{bind}^{residue}$ of the proposed catalytic residues (D223 and E286) are more favorable in the sucrose/WT and maltose/MT complexes than those of the maltose/WT and sucrose/MT complexes, respectively. These findings indicate that the catalytic residues favorably bind to sucrose in the WT active site and to maltose in the MT active site. This favorable binding between the catalytic residues and the substrates may also help facilitate the hydrolysis reaction, contributing to the substrate specificity of this enzyme.

Free energy components of the binding residues from three independent runs are shown in S14 and S15 Tables. Overall, the electrostatic interaction is the dominant contribution to the favorable binding of sucrose to the WT active site and maltose to the MT active site for most binding residues. For the proposed catalytic residues (D223 and E286), the electrostatic interaction is also a major contribution to the favorable binding of sucrose to the WT active site and of maltose to the MT active site. For the proposed sucrose preference residue (Y227), the van der Waals interaction makes a major contribution to the favorable binding of sucrose to the WT active site most likely due to the interaction between the aromatic ring of Y227 and sucrose. The strength of this van der Waals interaction is drastically reduced for the binding of sucrose to the MT active site. These results suggest the importance of the van der Waals interaction for Y227 to act as a sucrose preference residue. For the proposed maltose preference residue (H227), the electrostatic interaction makes a major contribution to the favorable binding of maltose to MT. The strength of this electrostatic interaction is drastically reduced for the binding of maltose to the WT active site. These findings suggest the importance of the electrostatic interaction for H227 acting as a maltose preference residue.

### Hydrogen bond interactions

To identify the hydrogen bonds important for substrate specificity, hydrogen bond occupations of all systems of the three independent runs were calculated (Table 2, S16 and S17 Tables). Strong, medium, and weak hydrogen bonds were defined as those with the hydrogen bond occupations of > 75%, 50–75%, and < 50%, respectively. Overall, the number of the hydrogen bonds of the sucrose/WT complex was greater than those of the maltose/WT complex, while the number of the hydrogen bonds of the sucrose/MT complex was less than that for the maltose/MT complex. These results support the rankings of the relative binding free energies and the experimental substrate specificities. Important binding residues contributing to the favorable binding of sucrose to the WT active site in all three independent runs are D81, D348, R413, and Y227 while those contributing to the favorable binding of maltose to the MT active site are D81, H227, D348 and R413. These binding residues have high hydrogen bond occupation and/or large number of hydrogen bonds. These results also support the roles of residue 227 acting as a substrate preference residue, Y227 acting as a sucrose preference residue in the WT active site and H227 acting as a maltose preference residue in the MT active site.

**Table 2:** Hydrogen bond occupations of sucrose/WT, maltose/WT, sucrose/MT and maltose/MT complexes from the 65–85 ns trajectories.

System	DONOR	ACCEPTORH	Occupancy* (%)
	res@atom	res@atom	
**Sucrose/WT complex**	D81@OD2	1GA563@H4O	92.71 (s)
	D81@OD1	1GA563@H3O	85.21 (s)
	H347@ND1	0CU564@H1O	19.89 (w)
	D348@OD2	0CU564@H4O	18.99 (w)
	D348@OD2	1GA563@H3O	71.25 (m)
	D348@OD1	1GA563@H2O	65.28 (m)
	D348@OD1	0CU564@H3O	55.02 (m)
	D348@OD2	1GA563@H2O	42.73 (w)
	D348@OD2	0CU564@H3O	34.13 (w)
	1GA563@O4	R413@HH11	82.2 (s)
	0CU564@O4	Y227@HH	77.73 (s)
	0CU564@O3	D348@H	15.24 (w)
**Maltose/WT complex**	D81@OD1	0GA565@H6O	88.81 (s)
	D81@OD2	0GA565@H4O	21.69 (w)
	D223@OD2	0GA565@H4O	82.91 (s)
	D348@OD1	0GA563@H2O	99.75 (s)
	D348@OD1	4GA564@H3O	99.00 (s)
	4GA564@O2	K312@HZ2	17.64 (w)
	0GA565@O2	R221@HH22	63.47 (m)
	0GA565@O2	H347@HE2	20.94 (w)
**Sucrose/MT complex**	D81@OD1	1GA563@H4O	74.36 (m)
	D81@OD1	1GA563@H3O	71.41 (m)
	D81@OD2	1GA563@H4O	49.28 (w)
	D81@OD2	1GA563@H3O	11.04 (w)
	D348@OD1	0CU564@H4O	15.94 (w)
	D348@OD2	0CU564@H4O	13.24 (w)
	1GA563@O4	Q399@HE21	15.64 (w)
	1GA563@O4	R413@HH12	64.22 (m)
**Maltose/MT complex**	D81@OD2	0GA565@H6O	80.51 (s)
	D81@OD1	0GA565@H4O	58.22 (m)
	D81@OD2	0GA565@H4O	57.27 (m)
	Q191@OE1	0GA565@H6O	14.99 (w)
	E286@OE2	4GA564@H2O	39.03 (w)
	D348@OD2	0GA565@H2O	98.72 (s)
	D348@OD1	0GA565@H3O	88.81 (s)
	D348@OD2	0GA565@H3O	15.74 (w)
	Q399@OE1	4GA564@H6O	10.14 (w)
	0GA564@O2	H227@HE2	88.48 (s)
	4GA564@O6	K254@HZ1	50.62 (m)
	0GA565@O4	R413@HH12	81.01 (s)

*Only hydrogen bonds with the occupations of more than 10% are shown: w = weak hydrogen bond, m = medium hydrogen bond, and s = strong hydrogen bond.

### The proximities between the atoms necessary for the hydrolysis reaction as well as between Y227/H227 and the furanose ring of sucrose/the pyranose ring of the glucosyl residue at the reducing end of maltose

**Fig 4:**
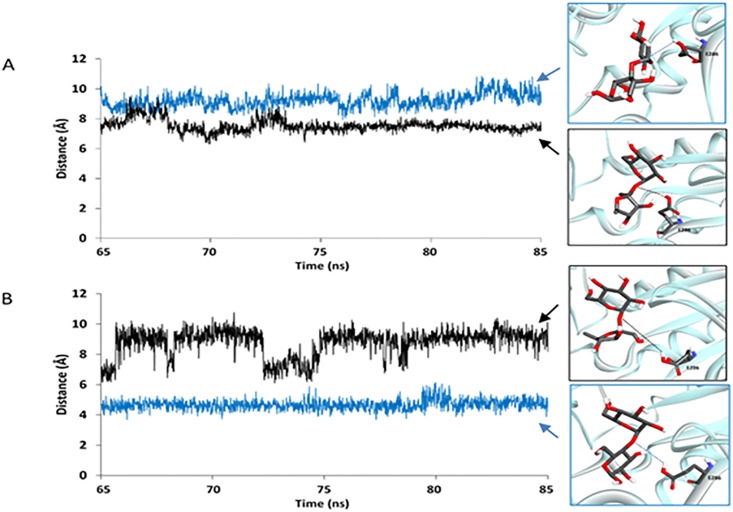
Distance between the proton of the carboxyl group of E286 and the oxygen atom of the glycosidic linkage of sucrose/maltose (d1): (A) sucrose/WT complex (black) and maltose/WT complex (blue). (B) sucrose/MT complex (black) and maltose/MT complex (blue).

With the assumption that this enzyme should be more likely to efficiently catalyze the hydrolysis reaction if the proton of the carboxyl group of catalytic E286 is closer to the oxygen atom of the glycosidic linkage of maltose/sucrose, the distance between the proton of the carboxyl group of catalytic E286 and the oxygen atom of the glycosidic linkage of maltose/sucrose (d1) was measured in all systems for three independent runs (Fig 4, S8 and S9 Figs). Our results show that d1 of the sucrose/WT complex is shorter than that of the maltose/WT complex, while d1 of the sucrose/MT complex is longer than that of the maltose/MT complex. These results suggest that WT may be more effective for catalyzing the hydrolysis of sucrose than for catalyzing the hydrolysis of maltose because on average the proton of the carboxyl group of catalytic E286 is closer to the oxygen atom of the glycosidic linkage of sucrose than that of maltose. In contrast, MT may be more effective for catalyzing the hydrolysis of maltose than for catalyzing the hydrolysis of sucrose because on average the proton of the carboxyl group of catalytic E286 is closer to the oxygen atom of the glycosidic linkage of maltose than that of sucrose.

**Fig 5:**
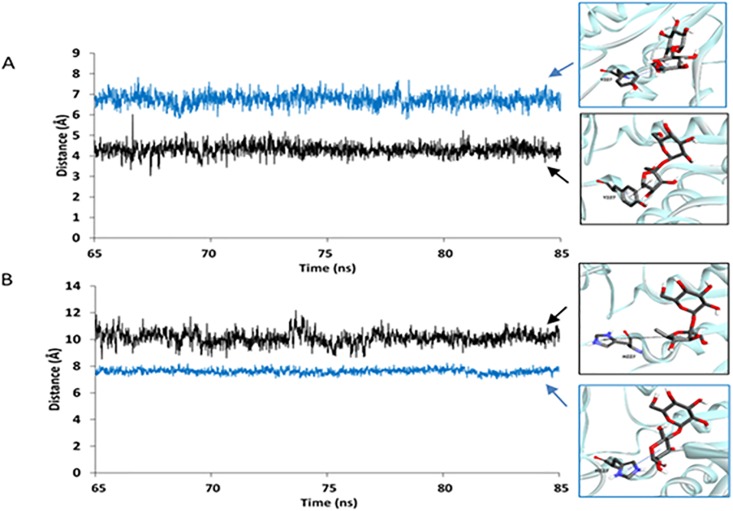
Distance between the center of mass of Y227/H227 and that of the furanose ring of sucrose/the pyranose ring of the glucosyl residue at the reducing end of maltose (d2): (A) sucrose/WT complex (black) and maltose/WT complex (blue), (B) sucrose/MT complex (black) and maltose/MT complex (blue).

With the assumption that the binding interaction between Y227/H227 and the fructosyl/glucosyl residue of sucrose/maltose is related to the substrate specificity of this enzyme, the distance between the center of mass of Y227/H227 and that of the furanose ring of sucrose/the pyranose ring of the glucosyl residue at the reducing end of maltose (d2) was measured in all systems for the three independent runs (Fig 5, S10 and S11 Figs). Our results show that d2 of the sucrose/WT complex is shorter than that of the maltose/WT complex, while d2 of the sucrose/MT complex is longer than that of the maltose/MT complex. These results suggest that WT may have better binding interaction between Y227 and the furanose ring of sucrose than that between Y227 and the pyranose ring of the glucosyl residue at the reducing end of maltose, supporting the role of Y227 as a sucrose preference residue. On the other hand, MT may have better binding interaction between H227 and the pyranose ring of the glucosyl residue at the reducing end of maltose than that between H227 and the furanose ring of sucrose. These results support the role of H227 as a maltose preference residue.

The Y227H mutation causes the change in d1 and d2 and, subsequently, the substrate specificity, most likely because H227 is smaller than Y227 and maltose is larger than sucrose; therefore, the active site of MT can more effectively bind and provide an appropriate distance for the hydrolysis of maltose than the active site of WT can. Moreover, the binding of maltose could cause unfavorable steric interactions with Y227 in the WT active site. As a result, the WT protein prefers binding sucrose to maltose, while MT prefers binding maltose to sucrose.

## Conclusions

The models of the sucrose/WT, maltose/WT, sucrose/MT, and maltose/MT complexes of *Apis mellifera* HBGase III were constructed and simulated at the experimental conditions [8] to gain insight into their binding dynamics, binding free energies and the mechanism of the changes of the substrate specificity of this enzyme due to the Y227 mutation. Based on their relative binding free energies, sucrose is preferred to maltose for WT binding, while maltose is preferred to sucrose for MT binding. The values of ${\Delta G}_{bind}^{residue}$ and hydrogen bond interaction results also support the rankings of the relative binding free energies. Moreover, the values of ${\Delta G}_{bind}^{residue}$ of Y227 and H227 support the previous study, which proposed that Y227/H227 were involved in the substrate preference for sucrose/maltose [8]. Our findings also suggest the importance of the van der Waals interaction for Y227 in acting as a sucrose preference residue and the electrostatic interaction for H227 in acting act as a maltose preference residue. Furthermore, the Y227H mutation caused changes in the proximities between the atoms necessary for the hydrolysis reaction (the distance between the proton of the carboxyl group of the catalytic E286 and the oxygen atom of the glycosidic linkage of sucrose/maltose) as well as between Y227/H227 and the furanose ring of sucrose/the pyranose ring of the glucosyl residue at the reducing end of maltose. These changes were most likely due to the smaller size of H227 compared to that of Y227 as well as the larger size of maltose compared to that of sucrose; therefore, the active site of MT could bind and more effectively provide an appropriate interatomic distance necessary for the hydrolysis reaction for maltose than for sucrose. Moreover, maltose could give rise to unfavorable steric interactions with Y227 in the WT active site. Our findings provide important insight into the binding of sucrose/maltose in the active site of *Apis mellifera* HBGase III and into the mechanism by which the Y227H mutation gives rise to the substrate specificity change. This knowledge could help in the future design of this enzyme for the increased production of desired products.

## Supporting information

S1 Fig Ramachandran plots of the homology model of HBGase III using *Erwinia rhapontici* isomaltulose synthase (4HPH) as a template. (TIF)

S2 Fig Superimpositions between the best docked conformation (pink) and crystal binding conformation (green) of maltose (3WY4) (left) with the RMSD value of 0.42 Å and those of sucrose (4HPH) (right) with the RMSD value of 0.43 Å. (TIF)

S3 Fig Best docked conformations: (A) sucrose/WT complex, (B) maltose/WT complex, (C) sucrose/MT complex, and (D) maltose/MT complex. Sucrose and maltose are shown in ball and stick representation. Binding residues are shown in licorice.
(TIF)

S4 Fig RMSD plots of the second independent runs: (A) sucrose/WT complex, (B) maltose/WT complex, (C) sucrose/MT complex, and (D) maltose/MT complex. (TIF)

S5 Fig RMSD plots of the third independent run: (A) sucrose/WT complex, (B) maltose/WT complex, (C) sucrose/MT complex, and (D) maltose/MT complex. (TIF)

S6 Fig Per-residue decomposition of binding free energy contributions of (A) sucrose/WT complex, (B) maltose/WT complex, (C) sucrose/MT complex, and (D) maltose/MT complex from the second independent run. (TIF)

S7 Fig Per-residue decomposition of binding free energy contributions of (A) sucrose/WT complex, (B) maltose/WT complex, (C) sucrose/MT complex, and (D) maltose/MT complex from the third independent run. (TIF)

S8 Fig Distance between the proton of the carboxyl group of E286 and the oxygen atom of the glycosidic linkage of sucrose/maltose (d1): (A) sucrose/WT complex (black) and maltose/WT complex (blue). (B) sucrose/MT complex (black) and maltose/MT complex (blue) from the second independent run.
(TIF)

S9 Fig Distance between the proton of the carboxyl group of E286 and the oxygen atom of the glycosidic linkage of sucrose/maltose (d1): (A) sucrose/WT complex (black) and maltose/WT complex (blue). (B) sucrose/MT complex (black) and maltose/MT complex (blue) from the third independent run.
(TIF)

S10 Fig Distance between the center of mass of Y227/H227 and the center of mass of the furanose ring of sucrose/ the pyranose ring of the glucosyl residue at the reducing end of maltose (d2) from the second independent run: (A) sucrose/WT complex (black) and maltose/WT complex (blue), (B) sucrose/MT complex (black) and maltose/MT complex (blue). (TIF)

S11 Fig Distance between the center of mass of Y227/H227 and the center of mass of the furanose ring of sucrose/ the pyranose ring of the glucosyl residue at the reducing end of maltose (d2) from the third independent run: (A) sucrose/WT complex (black) and maltose/WT complex (blue), (B) sucrose/MT complex (black) and maltose/MT complex (blue). (TIF)

S1 Table Affinity of docked conformations of sucrose/WT, maltose/WT, sucrose/MT and maltose/MT complexes. (DOCX)

S2 Table Binding free energies and their components for the second independent run of sucrose/WT, maltose/WT, sucrose/MT, and maltose/MT complexes. (DOCX)

S3 Table Binding free energies and their components for the third independent run of sucrose/WT, maltose/WT, sucrose/MT, and maltose/MT complexes. (DOCX)

S4 Table Energy contributions of the binding residues during 65 to 85 ns for the first independent run of the sucrose/WT complex. (DOCX)

S5 Table Energy contributions of the binding residues during 65 to 85 ns for the first independent run of the maltose/WT complex. (DOCX)

S6 Table Energy contributions of the binding residues during 65 to 85 ns for the first independent run of the sucrose/MT complex. (DOCX)

S7 Table Energy contributions of the binding residues during 65 to 85 ns for the first independent run of the maltose/MT complex. (DOCX)

S8 Table Energy contributions of the binding residues during 65 to 85 ns for the second independent run of the sucrose/WT complex. (DOCX)

S9 Table Energy contributions of the binding residues during 65 to 85 ns for the second independent run of the maltose/WT complex. (DOCX)

S10 Table Energy contributions of the binding residues during 65 to 85 ns for the second independent run of the sucrose/MT complex. (DOCX)

S11 Table Energy contributions of the binding residues during 65 to 85 ns for the second independent run of the maltose/MT complex. (DOCX)

S12 Table Energy contributions of the binding residues during 65 to 85 ns for the third independent run of the sucrose/WT complex. (DOCX)

S13 Table Energy contributions of the binding residues during 65 to 85 ns for the third independent run of the maltose/WT complex. (DOCX)

S14 Table Energy contributions of the binding residues during 65 to 85 ns for the third independent run of the sucrose/MT complex. (DOCX)

S15 Table Energy contributions of the binding residues during 65 to 85 ns for the third independent run of the maltose/MT complex. (DOCX)

S16 Table Hydrogen bond occupations of sucrose/WT, maltose/WT, sucrose/MT, and maltose/MT complexes from the 65 to 85 ns trajectories for the second independent run. (DOCX)

S17 Table Hydrogen bond occupations of sucrose/WT, maltose/WT, sucrose/MT and maltose/MT complex from the 65 to 85 ns trajectories for the third independent run. (DOCX)
