# Supplementary figures and images for: Insight into the substrate specificity change caused by the Y227H mutation of α-glucosidase III from the European honeybee (Apis mellifera) through molecular dynamics simulations
Source: PLoS One. 2018 Jun 4;13(6):e0198484. doi: 10.1371/journal.pone.0198484 (PMC5986129; doi:10.1371/journal.pone.0198484)

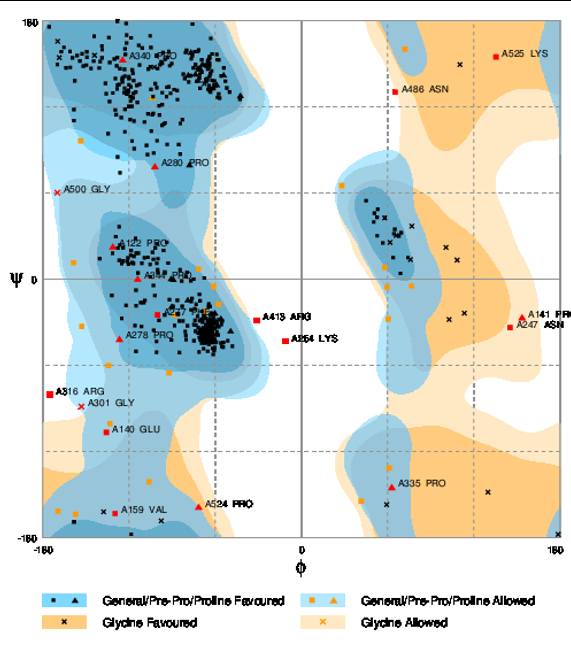

Supplement: S1 Fig — (TIF) [file pone.0198484.s001.tif]

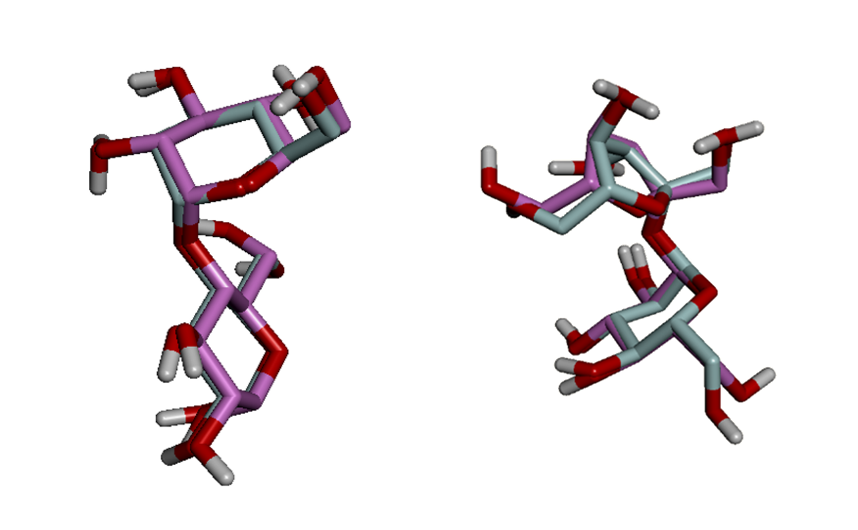

Supplement: S2 Fig — (TIF) [file pone.0198484.s002.tif]

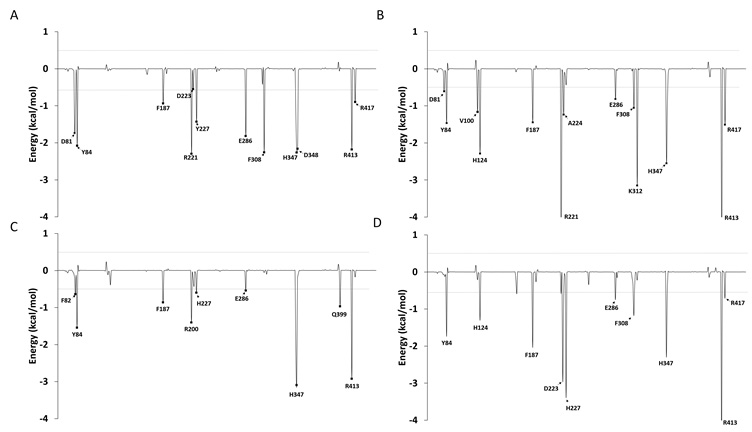

Supplement: S6 Fig — (TIF) [file pone.0198484.s006.tif]

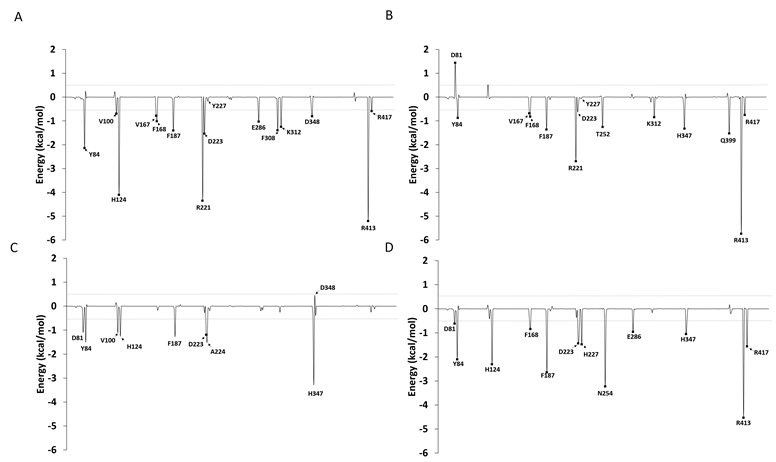

Supplement: S7 Fig — (TIF) [file pone.0198484.s007.tif]
