# Supplementary material for: Insight into the substrate specificity change caused by the Y227H mutation of α-glucosidase III from the European honeybee (Apis mellifera) through molecular dynamics simulations
Source: PLoS One. 2018 Jun 4;13(6):e0198484. doi: 10.1371/journal.pone.0198484 (PMC5986129; doi:10.1371/journal.pone.0198484)
Supplement: S1 Table — (DOCX) [file pone.0198484.s012.docx]

**S1 Table.** Affinity of docked conformations of sucrose/WT**,** maltose/WT**,** sucrose/MT **and** maltose/MT complexes.

| **System** | **Docked conformation** | **Affinity (kcal/mol)** |
| --- | --- | --- |
| **Sucrose/WT** | 1 | -7.2 |
|  | 2 | -6.5 |
|  | 3 | -6.4 |
|  | 4 | -5.9 |
|  | 5 | -5.9 |
|  | 6 | -5.8 |
|  | 7 | -5.8 |
|  | 8 | -5.7 |
|  | 9 | -5.5 |
| **Maltose/WT** | 1 | -7.4 |
|  | 2 | -7.2 |
|  | 3 | -6.8 |
|  | 4 | -6.8 |
|  | 5 | -6.7 |
|  | 6 | -6.6 |
|  | 7 | -6.2 |
|  | 8 | -6.1 |
|  | 9 | -6.1 |
| **Sucrose/MT** | 1 | -7.4 |
|  | 2 | -6.5 |
|  | 3 | -6.4 |
|  | 4 | -6.3 |
|  | 5 | -6.0 |
|  | 6 | -6.0 |
|  | 7 | -6.0 |
|  | 8 | -6.0 |
|  | 9 | -6.0 |
| **Maltose/MT** | 1 | -7.7 |
|  | 2 | -7.2 |
|  | 3 | -7.2 |
|  | 4 | -7.1 |
|  | 5 | -6.9 |
|  | 6 | -6.8 |
|  | 7 | -6.8 |
|  | 8 | -6.7 |
|  | 9 | -6.7 |
