# Supplementary material for: Insight into the substrate specificity change caused by the Y227H mutation of α-glucosidase III from the European honeybee (Apis mellifera) through molecular dynamics simulations
Source: PLoS One. 2018 Jun 4;13(6):e0198484. doi: 10.1371/journal.pone.0198484 (PMC5986129; doi:10.1371/journal.pone.0198484)
Supplement: S2 Table — (DOCX) [file pone.0198484.s013.docx]

**S2 Table.** Binding free energies and their components of the second independent run of sucrose/WT, maltose/WT, sucrose/MT, and maltose/MT complexes.

| System | Binding free energy and its compositions (kcal/mol) | | | | | | | | |
| --- | --- | --- | --- | --- | --- | --- | --- | --- | --- |
|  | **∆E_vdw_** | **∆E_ele_** | **∆G_pol_** | **∆G_np_** | | **^a)^∆G_solv_** | **-T∆S_tot_** | **^b)^∆G_bind_** | **s.e.m. of ∆G_bind_** |
| Sucrose/WT | -34.8 | -81.8 | 87.8 | -6.4 | 81.4 | | 25.1 | -10.1 | 1.8 |
| Maltose/WT | -27.9 | -70.7 | 81.4 | -5.1 | 76.3 | | 24.3 | -2.0 | 1.7 |
| Sucrose/MT | -33.5 | -102.0 | 104.1 | -6.3 | 97.7 | | 19.6 | -18.2 | 1.9 |
| Maltose/MT | -33.3 | -107.4 | 100.2 | -6.4 | 93.8 | | 18.7 | -28.3 | 1.9 |

**^a^∆G_solv_ = ∆G_pol_ + ∆G_np_**

**^b^∆G = ∆E_vdw_ + ∆E_ele_ + ∆G_solv_ - T∆S_tot_**
