# Supplementary material for: Insight into the substrate specificity change caused by the Y227H mutation of α-glucosidase III from the European honeybee (Apis mellifera) through molecular dynamics simulations
Source: PLoS One. 2018 Jun 4;13(6):e0198484. doi: 10.1371/journal.pone.0198484 (PMC5986129; doi:10.1371/journal.pone.0198484)
Supplement: S3 Table — (DOCX) [file pone.0198484.s014.docx]

**S3 Table.** Binding free energies and their components of the third independent run of sucrose/WT, maltose/WT, sucrose/MT, and maltose/MT complexes.

| System | Binding free energy and its compositions (kcal/mol) | | | | | | | | |
| --- | --- | --- | --- | --- | --- | --- | --- | --- | --- |
|  | **∆E_vdw_** | **∆E_ele_** | **∆G_pol_** | **∆G_np_** | | **^a)^∆G_solv_** | **-T∆S_tot_** | **^b)^∆G_bind_** | **s.e.m. of ∆G_bind_** |
| Sucrose/WT | -29.0 | -107.0 | 97.5 | -5.9 | 91.6 | | 25.4 | -19.0 | 1.7 |
| Maltose/WT | -29.7 | -84.9 | 93.3 | -5.7 | 88.6 | | 24.0 | -2.0 | 1.8 |
| Sucrose/MT | -23.8 | -82.1 | 90.1 | -4.7 | 85.4 | | 23.2 | 2.7 | 1.8 |
| Maltose/MT | -29.1 | -120.6 | 115.6 | -6.1 | 109.5 | | 25.3 | -14.9 | 2.0 |

**^a^∆G_solv_ = ∆G_pol_ + ∆G_np_**

**^b^∆G = ∆E_vdw_ + ∆E_ele_ + ∆G_solv_ - T∆S_tot_**
