# Supplementary material for: Insight into the substrate specificity change caused by the Y227H mutation of α-glucosidase III from the European honeybee (Apis mellifera) through molecular dynamics simulations
Source: PLoS One. 2018 Jun 4;13(6):e0198484. doi: 10.1371/journal.pone.0198484 (PMC5986129; doi:10.1371/journal.pone.0198484)
Supplement: S4 Table — (DOCX) [file pone.0198484.s015.docx]

**S4 Table.** Energy contributions of the binding residues during 65 to 85 ns of the first independent run of the sucrose/WT complex.

| Residue | Energy contribution (kcal/mol) of sucrose/WT complex | | | | | |
| --- | --- | --- | --- | --- | --- | --- |
|  | **Internal** | **van der Waals** | **Electrostatic** | **Polar solvation** | **Non-polar solvation** | **Total** |
| 81 | 0.00 | 0.00 | -5.91 | 6.98 | -0.03 | 1.03 |
| 82 | 0.00 | -0.05 | 0.03 | 0.03 | 0.00 | 0.01 |
| 84 | 0.00 | -2.37 | 0.58 | 0.35 | -0.11 | -1.54 |
| 121 | 0.00 | -0.21 | -0.01 | -0.01 | 0.00 | -0.22 |
| 124 | 0.00 | 0.05 | -3.56 | 0.38 | -0.02 | -3.15 |
| 167 | 0.00 | -0.97 | 0.00 | 0.10 | -0.14 | -1.00 |
| 168 | 0.00 | -1.36 | 0.22 | 0.17 | -0.13 | -1.10 |
| 187 | 0.00 | -1.42 | -0.03 | 0.41 | -0.11 | -1.16 |
| 191 | 0.00 | -0.54 | -0.05 | 0.07 | -0.01 | -0.53 |
| 221 | 0.00 | -0.42 | -0.92 | 0.46 | -0.03 | -0.91 |
| 223 | 0.00 | -0.42 | -5.33 | 5.26 | -0.11 | -0.60 |
| 224 | 0.00 | -0.62 | -0.35 | 0.01 | -0.01 | -0.97 |
| 227 | 0.00 | -2.48 | -1.00 | 2.40 | -0.13 | -1.21 |
| 252 | 0.00 | -0.04 | -0.06 | 0.09 | 0.00 | -0.01 |
| 254 | 0.00 | -0.74 | 0.73 | -0.56 | -0.15 | -0.71 |
| 286 | 0.00 | -0.60 | -1.48 | 0.68 | -0.12 | -1.53 |
| 308 | 0.00 | -0.67 | -0.11 | 0.23 | -0.05 | -0.61 |
| 312 | 0.00 | -0.63 | 0.16 | -0.25 | -0.14 | -0.85 |
| 347 | 0.00 | -0.41 | -0.78 | 0.63 | -0.02 | -0.60 |
| 348 | 0.00 | 0.40 | -14.26 | 13.86 | -0.15 | -0.15 |
| 399 | 0.00 | -0.12 | -0.10 | 0.18 | 0.00 | -0.04 |
| 417 | 0.00 | -0.22 | -0.64 | 0.00 | 0.00 | -0.86 |
