# Supplementary material for: Insight into the substrate specificity change caused by the Y227H mutation of α-glucosidase III from the European honeybee (Apis mellifera) through molecular dynamics simulations
Source: PLoS One. 2018 Jun 4;13(6):e0198484. doi: 10.1371/journal.pone.0198484 (PMC5986129; doi:10.1371/journal.pone.0198484)
Supplement: S5 Table — (DOCX) [file pone.0198484.s016.docx]

**S5 Table.** Energy contributions of the binding residues during 65 to 85 ns of the first independent run of the maltose/WT complex.

| Residue | Energy contribution (kcal/mol) of maltose/WT complex | | | | | |
| --- | --- | --- | --- | --- | --- | --- |
|  | **Internal** | **van der Waals** | **Electrostatic** | **Polar solvation** | **Non-polar solvation** | **Total** |
| 81 | 0.00 | -0.05 | -0.13 | 0.32 | 0.00 | 0.13 |
| 82 | 0.00 | -0.08 | 0.03 | 0.03 | 0.00 | -0.02 |
| 84 | 0.00 | -0.50 | 0.15 | 0.32 | -0.08 | -0.11 |
| 121 | 0.00 | -0.08 | -0.02 | 0.01 | 0.00 | -0.10 |
| 124 | 0.00 | -0.07 | -0.55 | 0.54 | -0.01 | -0.09 |
| 167 | 0.00 | -1.08 | -2.77 | 2.14 | -0.19 | -1.90 |
| 168 | 0.00 | -1.63 | -0.49 | 0.67 | -0.23 | -1.68 |
| 187 | 0.00 | -1.39 | -0.16 | 0.54 | -0.22 | -1.24 |
| 191 | 0.00 | -0.02 | 0.05 | -0.02 | 0.00 | 0.02 |
| 221 | 0.00 | -0.20 | -0.16 | 0.03 | 0.00 | -0.34 |
| 223 | 0.00 | -0.63 | -5.05 | 5.62 | -0.12 | -0.18 |
| 224 | 0.00 | -0.65 | -0.22 | 0.13 | -0.03 | -0.77 |
| 227 | 0.00 | -0.91 | -0.97 | 1.11 | -0.12 | -0.89 |
| 252 | 0.00 | -0.18 | 0.00 | 0.17 | -0.02 | -0.03 |
| 254 | 0.00 | -0.54 | -1.98 | 1.78 | -0.16 | -0.90 |
| 286 | 0.00 | 0.06 | -1.10 | 0.33 | -0.09 | -0.72 |
| 308 | 0.00 | -1.42 | -0.47 | 0.63 | -0.17 | -1.44 |
| 312 | 0.00 | -0.42 | -3.93 | 1.49 | -0.21 | -3.07 |
| 347 | 0.00 | -0.61 | -0.98 | 0.58 | -0.08 | -1.10 |
| 348 | 0.00 | 0.36 | -14.85 | 13.69 | -0.23 | -1.03 |
| 399 | 0.00 | -0.05 | -0.05 | 0.09 | 0.00 | -0.01 |
| 417 | 0.00 | -0.03 | 0.08 | -0.05 | 0.00 | 0.00 |
