# Supplementary material for: Insight into the substrate specificity change caused by the Y227H mutation of α-glucosidase III from the European honeybee (Apis mellifera) through molecular dynamics simulations
Source: PLoS One. 2018 Jun 4;13(6):e0198484. doi: 10.1371/journal.pone.0198484 (PMC5986129; doi:10.1371/journal.pone.0198484)
Supplement: S6 Table — (DOCX) [file pone.0198484.s017.docx]

**S6 Table.** Energy contributions of the binding residues during 65 to 85 ns of the first independent run of the sucrose/MT complex.

| Residue | Energy contribution (kcal/mol) of sucrose/MT complex | | | | | |
| --- | --- | --- | --- | --- | --- | --- |
|  | **Internal** | **van der Waals** | **Electrostatic** | **Polar solvation** | **Non-polar solvation** | **Total** |
| 81 | 0.00 | 0.77 | -17.15 | 15.71 | -0.08 | -0.75 |
| 82 | 0.00 | -0.10 | 0.06 | 0.02 | 0.00 | -0.02 |
| 84 | 0.00 | -2.66 | 0.68 | -0.03 | -0.19 | -2.20 |
| 121 | 0.00 | -0.22 | -0.11 | 0.13 | -0.01 | -0.21 |
| 124 | 0.00 | -0.46 | -4.82 | 2.93 | -0.07 | -2.42 |
| 167 | 0.00 | -0.45 | 0.00 | 0.05 | -0.08 | -0.48 |
| 168 | 0.00 | -0.77 | 0.03 | 0.06 | -0.09 | -0.77 |
| 187 | 0.00 | -2.05 | -0.33 | 0.54 | -0.30 | -2.14 |
| 191 | 0.00 | -0.39 | 0.55 | -0.22 | 0.00 | -0.05 |
| 221 | 0.00 | -0.20 | -0.66 | 0.61 | -0.01 | -0.26 |
| 223 | 0.00 | -0.30 | -8.74 | 8.98 | -0.28 | -0.35 |
| 224 | 0.00 | -0.47 | 0.17 | -0.15 | -0.13 | -0.58 |
| 227 | 0.00 | -0.08 | -0.37 | 0.47 | 0.00 | 0.02 |
| 252 | 0.00 | -0.01 | 0.03 | -0.01 | 0.00 | 0.01 |
| 254 | 0.00 | -0.11 | -0.50 | 0.76 | -0.04 | 0.10 |
| 286 | 0.00 | -0.13 | 0.14 | -0.08 | 0.00 | -0.08 |
| 308 | 0.00 | -0.10 | 0.01 | 0.03 | -0.01 | -0.07 |
| 312 | 0.00 | -0.04 | 0.13 | -0.06 | -0.01 | 0.02 |
| 347 | 0.00 | -0.83 | -2.23 | 0.86 | -0.08 | -2.28 |
| 348 | 0.00 | -0.64 | -10.43 | 12.23 | -0.28 | 0.87 |
| 399 | 0.00 | -0.10 | 0.19 | -0.16 | 0.00 | -0.08 |
| 417 | 0.00 | -0.11 | 0.30 | -0.42 | -0.03 | -0.26 |
