# Supplementary material for: Insight into the substrate specificity change caused by the Y227H mutation of α-glucosidase III from the European honeybee (Apis mellifera) through molecular dynamics simulations
Source: PLoS One. 2018 Jun 4;13(6):e0198484. doi: 10.1371/journal.pone.0198484 (PMC5986129; doi:10.1371/journal.pone.0198484)
Supplement: S7 Table — (DOCX) [file pone.0198484.s018.docx]

**S7 Table.** Energy contributions of the binding residues during 65 to 85 ns of the first independent run of the maltose/MT complex.

| Residue | Energy contribution (kcal/mol) of maltose/MT complex | | | | | |
| --- | --- | --- | --- | --- | --- | --- |
|  | **Internal** | **van der Waals** | **Electrostatic** | **Polar solvation** | **Non-polar solvation** | **Total** |
| 81 | 0.00 | 0.80 | -16.12 | 14.30 | -0.07 | -1.09 |
| 82 | 0.00 | -0.28 | -0.01 | 0.18 | 0.00 | -0.11 |
| 84 | 0.00 | -1.91 | 0.30 | -0.05 | -0.09 | -1.75 |
| 121 | 0.00 | -0.50 | -0.14 | 0.13 | -0.03 | -0.54 |
| 124 | 0.00 | -0.06 | -6.13 | 3.26 | -0.04 | -2.97 |
| 167 | 0.00 | -0.07 | -0.04 | 0.05 | 0.00 | -0.06 |
| 168 | 0.00 | -0.02 | 0.00 | 0.01 | 0.00 | -0.01 |
| 187 | 0.00 | -2.29 | -0.05 | 0.13 | -0.20 | -2.40 |
| 191 | 0.00 | -0.62 | 0.93 | -0.28 | 0.00 | 0.02 |
| 221 | 0.00 | -0.26 | 2.70 | -2.86 | 0.00 | -0.42 |
| 223 | 0.00 | -0.08 | -15.04 | 12.46 | -0.15 | -2.82 |
| 224 | 0.00 | -1.10 | -0.14 | -0.32 | -0.09 | -1.65 |
| 227 | 0.00 | -0.12 | -9.02 | 5.92 | -0.08 | -3.30 |
| 252 | 0.00 | -0.04 | 0.05 | 0.00 | 0.00 | 0.01 |
| 254 | 0.00 | -1.60 | -4.45 | 2.59 | -0.18 | -3.64 |
| 286 | 0.00 | 1.80 | -1.04 | -0.86 | -0.10 | -0.20 |
| 308 | 0.00 | -0.82 | -0.08 | 0.39 | -0.18 | -0.71 |
| 312 | 0.00 | -0.02 | 0.21 | -0.19 | 0.00 | 0.01 |
| 347 | 0.00 | -0.56 | -1.11 | 0.10 | -0.03 | -1.59 |
| 348 | 0.00 | -0.62 | -4.82 | 6.88 | -0.14 | 1.31 |
| 399 | 0.00 | -0.77 | -0.15 | 0.18 | -0.09 | -0.83 |
| 417 | 0.00 | -0.16 | -0.24 | -0.13 | 0.00 | -0.53 |
