# Supplementary material for: Insight into the substrate specificity change caused by the Y227H mutation of α-glucosidase III from the European honeybee (Apis mellifera) through molecular dynamics simulations
Source: PLoS One. 2018 Jun 4;13(6):e0198484. doi: 10.1371/journal.pone.0198484 (PMC5986129; doi:10.1371/journal.pone.0198484)
Supplement: S8 Table — (DOCX) [file pone.0198484.s019.docx]

**S8 Table.** Energy contributions of the binding residues during 65 to 85 ns of the second independent run of the sucrose/WT complex.

| Residue | Energy contribution (kcal/mol) of sucrose/WT complex | | | | | |
| --- | --- | --- | --- | --- | --- | --- |
|  | **Internal** | **van der Waals** | **Electrostatic** | **Polar solvation** | **Non-polar solvation** | **Total** |
| 81 | 0.00 | 0.22 | -17.32 | 15.44 | -0.08 | -1.74 |
| 82 | 0.00 | -0.94 | -0.34 | 0.37 | -0.07 | -0.97 |
| 84 | 0.00 | -1.97 | -0.02 | 0.09 | -0.19 | -2.08 |
| 121 | 0.00 | -0.07 | 0.00 | 0.01 | 0.00 | -0.06 |
| 124 | 0.00 | -0.12 | 0.97 | -0.88 | -0.01 | -0.04 |
| 167 | 0.00 | -0.11 | 0.00 | 0.03 | -0.02 | -0.10 |
| 168 | 0.00 | -0.19 | 0.08 | -0.02 | -0.02 | -0.15 |
| 187 | 0.00 | -0.72 | -0.01 | 0.00 | -0.21 | -0.94 |
| 191 | 0.00 | -0.12 | 0.19 | -0.20 | 0.00 | -0.13 |
| 221 | 0.00 | -0.81 | -2.01 | 0.67 | -0.15 | -2.30 |
| 223 | 0.00 | -0.30 | -5.27 | 5.19 | -0.16 | -0.55 |
| 224 | 0.00 | -0.07 | -0.13 | 0.19 | 0.00 | -0.01 |
| 227 | 0.00 | -1.46 | -0.14 | 0.18 | 0.00 | -1.43 |
| 252 | 0.00 | -0.03 | 0.03 | 0.00 | 0.00 | 0.00 |
| 254 | 0.00 | -0.11 | -1.95 | 2.07 | -0.06 | -0.05 |
| 286 | 0.00 | -0.43 | -1.36 | 0.01 | -0.04 | -1.82 |
| 308 | 0.00 | -2.00 | -0.80 | 0.81 | -0.27 | -2.26 |
| 312 | 0.00 | -0.02 | 0.01 | 0.03 | 0.00 | 0.02 |
| 347 | 0.00 | -1.84 | -1.08 | 0.83 | -0.17 | -2.26 |
| 348 | 0.00 | -1.53 | -15.74 | 15.38 | -0.28 | -2.16 |
| 399 | 0.00 | -0.06 | -0.14 | 0.23 | 0.00 | 0.03 |
| 417 | 0.00 | -0.27 | -1.03 | 0.42 | -0.02 | -0.90 |
