# Supplementary material for: Insight into the substrate specificity change caused by the Y227H mutation of α-glucosidase III from the European honeybee (Apis mellifera) through molecular dynamics simulations
Source: PLoS One. 2018 Jun 4;13(6):e0198484. doi: 10.1371/journal.pone.0198484 (PMC5986129; doi:10.1371/journal.pone.0198484)
Supplement: S9 Table — (DOCX) [file pone.0198484.s020.docx]

**S9 Table.** Energy contributions of the binding residues during 65 to 85 ns of the second independent run of the maltose/WT complex.

| Residue | Energy contribution (kcal/mol) of maltose/WT complex | | | | | |
| --- | --- | --- | --- | --- | --- | --- |
|  | **Internal** | **van der Waals** | **Electrostatic** | **Polar solvation** | **Non-polar solvation** | **Total** |
| 81 | 0.00 | 0.37 | -15.22 | 14.31 | -0.07 | -0.61 |
| 82 | 0.00 | -0.13 | 0.06 | 0.03 | 0.00 | -0.04 |
| 84 | 0.00 | -2.78 | 1.06 | 0.33 | -0.07 | -1.47 |
| 121 | 0.00 | -1.05 | -0.06 | 0.01 | -0.07 | -1.17 |
| 124 | 0.00 | -0.43 | -3.87 | 2.04 | -0.03 | -2.29 |
| 167 | 0.00 | -0.10 | 0.05 | -0.03 | 0.00 | -0.08 |
| 168 | 0.00 | -0.04 | 0.03 | -0.01 | 0.00 | -0.02 |
| 187 | 0.00 | -1.35 | -0.23 | 0.31 | -0.18 | -1.45 |
| 191 | 0.00 | -0.55 | 1.50 | -0.85 | -0.01 | 0.08 |
| 221 | 0.00 | -0.79 | -6.65 | 1.47 | -0.08 | -6.05 |
| 223 | 0.00 | 0.12 | -2.23 | 2.11 | -0.11 | -0.11 |
| 224 | 0.00 | -0.86 | -0.28 | -0.05 | -0.06 | -1.24 |
| 227 | 0.00 | -0.47 | -0.38 | 0.49 | -0.08 | -0.43 |
| 252 | 0.00 | -0.01 | -0.03 | 0.05 | 0.00 | 0.01 |
| 254 | 0.00 | -0.20 | 1.29 | -1.02 | -0.10 | -0.03 |
| 286 | 0.00 | -0.20 | -1.83 | -0.36 | -0.10 | -0.82 |
| 308 | 0.00 | -1.22 | -0.56 | 0.82 | -0.10 | -1.06 |
| 312 | 0.00 | -0.24 | -6.99 | 4.32 | -0.25 | -3.15 |
| 347 | 0.00 | -0.88 | -2.22 | 0.56 | -0.01 | -2.55 |
| 348 | 0.00 | 1.15 | -23.07 | 19.93 | -0.17 | -2.16 |
| 399 | 0.00 | -0.34 | -0.08 | 0.24 | -0.03 | -0.22 |
| 417 | 0.00 | -0.37 | 0.38 | -1.52 | 0.00 | -1.51 |
