# Supplementary material for: Insight into the substrate specificity change caused by the Y227H mutation of α-glucosidase III from the European honeybee (Apis mellifera) through molecular dynamics simulations
Source: PLoS One. 2018 Jun 4;13(6):e0198484. doi: 10.1371/journal.pone.0198484 (PMC5986129; doi:10.1371/journal.pone.0198484)
Supplement: S10 Table — (DOCX) [file pone.0198484.s021.docx]

**S10 Table.** Energy contributions of the binding residues during 65 to 85 ns of the second independent run of the sucrose/MT complex.

| Residue | Energy contribution (kcal/mol) of sucrose/MT complex | | | | | |
| --- | --- | --- | --- | --- | --- | --- |
|  | **Internal** | **van der Waals** | **Electrostatic** | **Polar solvation** | **Non-polar solvation** | **Total** |
| 81 | 0.00 | 0.29 | -17.71 | 17.32 | -0.09 | -0.19 |
| 82 | 0.00 | -0.82 | -0.17 | 0.38 | -0.03 | -0.64 |
| 84 | 0.00 | -2.00 | -0.03 | 0.58 | -0.09 | -1.54 |
| 121 | 0.00 | -0.14 | 0.00 | 0.02 | 0.00 | -0.12 |
| 124 | 0.00 | -0.31 | 0.88 | -0.91 | -0.06 | -0.39 |
| 167 | 0.00 | -0.03 | 0.05 | -0.04 | 0.00 | -0.02 |
| 168 | 0.00 | -0.01 | 0.02 | -0.01 | 0.00 | 0.00 |
| 187 | 0.00 | -1.11 | -0.07 | 0.46 | -0.14 | -0.86 |
| 191 | 0.00 | -0.12 | -0.36 | 0.49 | 0.00 | 0.02 |
| 221 | 0.00 | -0.41 | -2.16 | 1.24 | -0.08 | -1.41 |
| 223 | 0.00 | -0.27 | -6.67 | 6.87 | -0.15 | -0.22 |
| 224 | 0.00 | -0.41 | 0.27 | -0.23 | -0.06 | -0.43 |
| 227 | 0.00 | -0.44 | -2.45 | 2.41 | -0.13 | -0.60 |
| 252 | 0.00 | -0.01 | -0.07 | 0.09 | 0.00 | 0.01 |
| 254 | 0.00 | -0.14 | 0.92 | -0.77 | -0.01 | 0.00 |
| 286 | 0.00 | -1.08 | 0.17 | 0.55 | -0.18 | -0.54 |
| 308 | 0.00 | -0.11 | -0.04 | 0.09 | 0.00 | -0.06 |
| 312 | 0.00 | -0.01 | -0.18 | 0.19 | 0.00 | 0.00 |
| 347 | 0.00 | -2.03 | -3.55 | 2.74 | -0.25 | -3.09 |
| 348 | 0.00 | -1.10 | -3.29 | 4.37 | -0.21 | -0.22 |
| 399 | 0.00 | -0.68 | -0.05 | -0.15 | -0.09 | -0.97 |
| 417 | 0.00 | -0.10 | 0.56 | -0.63 | 0.00 | -0.18 |
