# Supplementary material for: Insight into the substrate specificity change caused by the Y227H mutation of α-glucosidase III from the European honeybee (Apis mellifera) through molecular dynamics simulations
Source: PLoS One. 2018 Jun 4;13(6):e0198484. doi: 10.1371/journal.pone.0198484 (PMC5986129; doi:10.1371/journal.pone.0198484)
Supplement: S11 Table — (DOCX) [file pone.0198484.s022.docx]

**S11 Table.** Energy contributions of the binding residues during 65 to 85 ns of the second independent run of the maltose/MT complex.

| Residue | Energy contribution (kcal/mol) of maltose/MT complex | | | | | |
| --- | --- | --- | --- | --- | --- | --- |
|  | **Internal** | **van der Waals** | **Electrostatic** | **Polar solvation** | **Non-polar solvation** | **Total** |
| 81 | 0.00 | 0.53 | -14.71 | 14.18 | -0.07 | -0.07 |
| 82 | 0.00 | -0.33 | 0.00 | 0.22 | 0.00 | -0.12 |
| 84 | 0.00 | -1.79 | 0.28 | -0.10 | -0.14 | -1.74 |
| 121 | 0.00 | -0.20 | -0.07 | 0.07 | -0.02 | -0.21 |
| 124 | 0.00 | -0.47 | -3.31 | 2.53 | -0.06 | -1.31 |
| 167 | 0.00 | -0.24 | -0.03 | 0.05 | -0.01 | -0.24 |
| 168 | 0.00 | -0.58 | 0.11 | -0.07 | -0.06 | -0.59 |
| 187 | 0.00 | -1.80 | -0.17 | 0.21 | -0.27 | -2.04 |
| 191 | 0.00 | -0.23 | -0.73 | 0.70 | -0.01 | -0.27 |
| 221 | 0.00 | -0.23 | -1.65 | 1.33 | -0.04 | -0.59 |
| 223 | 0.00 | -0.60 | -12.07 | 9.94 | -0.24 | -2.96 |
| 224 | 0.00 | -0.51 | 0.20 | -0.33 | -0.06 | -0.70 |
| 227 | 0.00 | -0.44 | -3.89 | 1.08 | -0.06 | -3.31 |
| 252 | 0.00 | -0.01 | 0.06 | -0.05 | 0.00 | 0.00 |
| 254 | 0.00 | -0.18 | -1.47 | 1.37 | -0.06 | -0.35 |
| 286 | 0.00 | 0.31 | -0.68 | -0.26 | -0.12 | -0.75 |
| 308 | 0.00 | -0.66 | -0.71 | 0.28 | -0.09 | -1.18 |
| 312 | 0.00 | -0.34 | 2.17 | -1.98 | -0.07 | -0.22 |
| 347 | 0.00 | -0.65 | -2.07 | 0.47 | -0.05 | -2.29 |
| 348 | 0.00 | 0.67 | -15.48 | 14.33 | -0.15 | -0.63 |
| 399 | 0.00 | -0.28 | -0.31 | 0.54 | -0.02 | -0.06 |
| 417 | 0.00 | -0.19 | 0.62 | -1.14 | 0.00 | -0.71 |
