# Supplementary material for: Insight into the substrate specificity change caused by the Y227H mutation of α-glucosidase III from the European honeybee (Apis mellifera) through molecular dynamics simulations
Source: PLoS One. 2018 Jun 4;13(6):e0198484. doi: 10.1371/journal.pone.0198484 (PMC5986129; doi:10.1371/journal.pone.0198484)
Supplement: S12 Table — (DOCX) [file pone.0198484.s023.docx]

**S12 Table.** Energy contributions of the binding residues during 65 to 85 ns of the third independent run of the sucrose/WT complex.

| Residue | Energy contribution (kcal/mol) of sucrose/WT complex | | | | | |
| --- | --- | --- | --- | --- | --- | --- |
|  | **Internal** | **van der Waals** | **Electrostatic** | **Polar solvation** | **Non-polar solvation** | **Total** |
| 81 | 0.00 | 0.49 | -14.61 | 14.12 | -0.05 | -0.06 |
| 82 | 0.00 | -0.25 | -0.01 | 0.18 | 0.00 | -0.08 |
| 84 | 0.00 | -2.54 | 0.09 | 0.45 | -0.14 | -2.14 |
| 121 | 0.00 | -0.62 | -0.08 | 0.04 | -0.05 | -0.70 |
| 124 | 0.00 | -0.58 | -7.66 | 4.20 | -0.06 | -4.10 |
| 167 | 0.00 | -0.74 | 0.04 | 0.07 | -0.15 | -0.79 |
| 168 | 0.00 | -0.97 | -0.13 | 0.20 | -0.12 | -1.02 |
| 187 | 0.00 | -1.90 | 0.76 | -0.06 | -0.20 | -1.40 |
| 191 | 0.00 | -0.44 | 0.51 | -0.07 | -0.01 | -0.01 |
| 221 | 0.00 | -0.50 | -3.06 | -0.75 | -0.05 | -4.36 |
| 223 | 0.00 | -0.31 | -5.65 | 4.55 | -0.13 | -1.53 |
| 224 | 0.00 | -0.52 | 0.03 | 0.05 | -0.05 | -0.49 |
| 227 | 0.00 | -0.21 | -0.19 | 0.27 | -0.05 | -0.17 |
| 252 | 0.00 | -0.11 | 0.17 | -0.12 | -0.03 | -0.08 |
| 254 | 0.00 | -0.10 | -1.46 | 1.49 | -0.05 | -0.12 |
| 286 | 0.00 | -0.20 | -4.66 | 3.99 | -0.15 | -1.03 |
| 308 | 0.00 | -1.47 | -0.26 | 0.50 | -0.16 | -1.39 |
| 312 | 0.00 | -0.68 | 2.21 | -2.68 | -0.10 | -1.25 |
| 347 | 0.00 | -0.44 | -0.04 | 0.06 | 0.00 | -0.43 |
| 348 | 0.00 | 1.26 | -15.58 | 13.55 | -0.04 | -0.81 |
| 399 | 0.00 | -0.20 | -0.26 | 0.46 | 0.00 | -0.01 |
| 417 | 0.00 | -0.14 | 0.27 | -0.72 | 0.00 | -0.59 |
