# Supplementary material for: Insight into the substrate specificity change caused by the Y227H mutation of α-glucosidase III from the European honeybee (Apis mellifera) through molecular dynamics simulations
Source: PLoS One. 2018 Jun 4;13(6):e0198484. doi: 10.1371/journal.pone.0198484 (PMC5986129; doi:10.1371/journal.pone.0198484)
Supplement: S13 Table — (DOCX) [file pone.0198484.s024.docx]

**S13 Table.** Energy contributions of the binding residues during 65 to 85 ns of the third independent run of the maltose/WT complex.

| Residue | Energy contribution (kcal/mol) of maltose/WT complex | | | | | |
| --- | --- | --- | --- | --- | --- | --- |
|  | **Internal** | **van der Waals** | **Electrostatic** | **Polar solvation** | **Non-polar solvation** | **Total** |
| 81 | 0.00 | 0.18 | -5.83 | 7.14 | -0.04 | 1.44 |
| 82 | 0.00 | -0.06 | 0.03 | 0.02 | 0.00 | -0.01 |
| 84 | 0.00 | -1.76 | -0.20 | 1.18 | -0.09 | -0.88 |
| 121 | 0.00 | -0.09 | 0.11 | -0.09 | 0.00 | -0.07 |
| 124 | 0.00 | -0.10 | 2.26 | -2.13 | -0.02 | 0.01 |
| 167 | 0.00 | -0.65 | -0.05 | 0.06 | -0.04 | -0.68 |
| 168 | 0.00 | -0.87 | -0.01 | 0.13 | -0.08 | -0.83 |
| 187 | 0.00 | -1.25 | -0.13 | 0.23 | -0.21 | -1.36 |
| 191 | 0.00 | -0.11 | -0.13 | 0.13 | 0.00 | -0.11 |
| 221 | 0.00 | -0.65 | -2.96 | 0.99 | -0.07 | -2.70 |
| 223 | 0.00 | 0.33 | -14.42 | 13.62 | -0.16 | -0.63 |
| 224 | 0.00 | -0.29 | 0.14 | -0.14 | -0.02 | -0.31 |
| 227 | 0.00 | -0.26 | 0.13 | 0.08 | -0.02 | -0.07 |
| 252 | 0.00 | -1.13 | 0.10 | 0.09 | -0.32 | -1.26 |
| 254 | 0.00 | -0.07 | -0.95 | 1.02 | 0.00 | -0.01 |
| 286 | 0.00 | -0.15 | -0.46 | 0.88 | -0.14 | 0.13 |
| 308 | 0.00 | -0.29 | -0.09 | 0.24 | -0.07 | -0.21 |
| 312 | 0.00 | -0.21 | -2.85 | 2.33 | -0.12 | -0.84 |
| 347 | 0.00 | -0.76 | -0.87 | 0.33 | -0.03 | -1.33 |
| 348 | 0.00 | -2.13 | -5.11 | 7.66 | -0.30 | 0.11 |
| 399 | 0.00 | -0.43 | -4.80 | 3.86 | -0.16 | -1.53 |
| 417 | 0.00 | -0.26 | -1.67 | 1.18 | 0.00 | -0.75 |
