# Supplementary material for: Insight into the substrate specificity change caused by the Y227H mutation of α-glucosidase III from the European honeybee (Apis mellifera) through molecular dynamics simulations
Source: PLoS One. 2018 Jun 4;13(6):e0198484. doi: 10.1371/journal.pone.0198484 (PMC5986129; doi:10.1371/journal.pone.0198484)
Supplement: S14 Table — (DOCX) [file pone.0198484.s025.docx]

**S14 Table.** Energy contributions of the binding residues during 65 to 85 ns of the third independent run of the sucrose/MT complex.

| Residue | Energy contribution (kcal/mol) of sucrose/MT complex | | | | | |
| --- | --- | --- | --- | --- | --- | --- |
|  | **Internal** | **van der Waals** | **Electrostatic** | **Polar solvation** | **Non-polar solvation** | **Total** |
| 81 | 0.00 | 0.73 | -19.93 | 18.30 | -0.19 | -1.10 |
| 82 | 0.00 | -0.59 | 0.03 | 0.19 | -0.04 | -0.41 |
| 84 | 0.00 | -1.45 | 0.26 | -0.24 | -0.07 | -1.50 |
| 121 | 0.00 | -0.97 | -0.15 | 0.13 | -0.12 | -1.11 |
| 124 | 0.00 | -0.25 | 0.44 | -1.42 | -0.03 | -1.26 |
| 167 | 0.00 | -0.15 | 0.05 | -0.04 | -0.03 | -0.17 |
| 168 | 0.00 | -0.09 | 0.08 | -0.04 | -0.01 | -0.06 |
| 187 | 0.00 | -1.28 | -0.02 | 0.33 | -0.31 | -1.28 |
| 191 | 0.00 | -0.11 | -0.26 | 0.39 | 0.00 | 0.02 |
| 221 | 0.00 | -0.16 | 1.03 | -1.15 | 0.00 | -0.28 |
| 223 | 0.00 | -0.47 | -7.88 | 7.30 | -0.14 | -1.19 |
| 224 | 0.00 | -1.28 | 0.31 | -0.28 | -0.23 | -1.49 |
| 227 | 0.00 | -0.20 | -1.12 | 1.17 | -0.03 | -0.19 |
| 252 | 0.00 | -0.02 | 0.10 | -0.09 | 0.00 | 0.00 |
| 254 | 0.00 | -0.10 | -1.26 | 1.37 | -0.04 | -0.03 |
| 286 | 0.00 | -0.41 | 1.49 | -1.23 | -0.04 | -0.20 |
| 308 | 0.00 | -0.28 | 0.02 | 0.06 | -0.05 | -0.25 |
| 312 | 0.00 | -0.09 | -1.20 | 1.29 | -0.03 | -0.03 |
| 347 | 0.00 | -0.47 | -3.71 | 0.99 | -0.09 | -3.29 |
| 348 | 0.00 | -1.24 | -6.96 | 8.93 | -0.35 | 0.38 |
| 399 | 0.00 | -0.02 | -0.02 | 0.05 | 0.00 | 0.01 |
| 417 | 0.00 | -0.08 | 0.11 | -0.13 | -0.01 | -0.11 |
