# Supplementary material for: Insight into the substrate specificity change caused by the Y227H mutation of α-glucosidase III from the European honeybee (Apis mellifera) through molecular dynamics simulations
Source: PLoS One. 2018 Jun 4;13(6):e0198484. doi: 10.1371/journal.pone.0198484 (PMC5986129; doi:10.1371/journal.pone.0198484)
Supplement: S15 Table — (DOCX) [file pone.0198484.s026.docx]

**S15 Table.** Energy contributions of the binding residues during 65 to 85 ns of the third independent run of the maltose/MT complex.

| Residue | Energy contribution (kcal/mol) of maltose/MT complex | | | | | |
| --- | --- | --- | --- | --- | --- | --- |
|  | **Internal** | **van der Waals** | **Electrostatic** | **Polar solvation** | **Non-polar solvation** | **Total** |
| 81 | 0.00 | 0.69 | -16.14 | 14.89 | -0.06 | -0.62 |
| 82 | 0.00 | -0.32 | 0.04 | 0.21 | 0.00 | -0.07 |
| 84 | 0.00 | -2.38 | 0.60 | -0.19 | -0.14 | -2.11 |
| 121 | 0.00 | -0.37 | -0.16 | 0.15 | -0.04 | -0.42 |
| 124 | 0.00 | -0.41 | -5.57 | 3.74 | -0.06 | -2.31 |
| 167 | 0.00 | -0.50 | -0.06 | 0.07 | -0.02 | -0.51 |
| 168 | 0.00 | -0.84 | 0.12 | -0.04 | -0.09 | -0.85 |
| 187 | 0.00 | -2.36 | -0.36 | 0.33 | -0.25 | -2.64 |
| 191 | 0.00 | -0.42 | 0.79 | -0.46 | 0.00 | -0.09 |
| 221 | 0.00 | -0.18 | -0.01 | -0.15 | -0.02 | -0.37 |
| 223 | 0.00 | -0.44 | -10.58 | 9.84 | -0.26 | -1.44 |
| 224 | 0.00 | -0.83 | 0.01 | -0.25 | -0.08 | -1.15 |
| 227 | 0.00 | -0.32 | -3.92 | 2.84 | -0.09 | -1.48 |
| 252 | 0.00 | -0.04 | 0.12 | -0.06 | 0.00 | 0.02 |
| 254 | 0.00 | -1.35 | -4.95 | 3.35 | -0.27 | -3.23 |
| 286 | 0.00 | 0.25 | -0.91 | -0.29 | -0.01 | -0.96 |
| 308 | 0.00 | -0.22 | -0.04 | 0.14 | -0.04 | -0.16 |
| 312 | 0.00 | -0.02 | 0.16 | -0.14 | 0.00 | 0.00 |
| 347 | 0.00 | -0.58 | -1.30 | 0.85 | -0.02 | -1.05 |
| 348 | 0.00 | 0.73 | -18.16 | 17.17 | -0.24 | -0.49 |
| 399 | 0.00 | -0.31 | -0.89 | 1.09 | -0.03 | -0.15 |
| 417 | 0.00 | -0.38 | 0.72 | -1.90 | 0.00 | -1.57 |
