# Supplementary material for: Insight into the substrate specificity change caused by the Y227H mutation of α-glucosidase III from the European honeybee (Apis mellifera) through molecular dynamics simulations
Source: PLoS One. 2018 Jun 4;13(6):e0198484. doi: 10.1371/journal.pone.0198484 (PMC5986129; doi:10.1371/journal.pone.0198484)
Supplement: S16 Table — (DOCX) [file pone.0198484.s027.docx]

**S16 Table.** Hydrogen bond occupations of the second independent run of sucrose/WT, maltose/WT, sucrose/MT, and maltose/MT complexes from the 65 to 85 ns trajectory.

| System | DONOR | ACCEPTORH | Occupancy* (%) |
| --- | --- | --- | --- |
|  | **res@atom** | **res@atom** |  |
| Sucrose/WT complex | D81@OD2 | 1GA563@H4O | 67.27 (m) |
|  | D81@OD2 | 1GA563@H6O | 66.82 (m) |
|  | D81@OD1 | 1GA563@H4O | 40.03 (w) |
|  | Q191@OE1 | 1GA563@H6O | 28.24 (w) |
|  | Y227@OH | 0GA563@H3O | 88.87 (s) |
|  | D348@OD1 | 1GA563@H2O | 99.75 (s) |
|  | D348@OD1 | 1GA563@H3O | 95.05 (s) |
|  | D348@OD2 | 1GA563@H3O | 16.29 (w) |
|  | 1GA563@O4 | R413@HH11 | 42.03 (w) |
|  | 1GA563@O6 | H124@HE2 | 26.09 (w) |
|  | 0CU564@O3 | E286@HE2 | 96.15 (s) |
|  | 0CU564@O4 | Y227@HH | 48.61 (w) |
| Maltose/WT complex | D81@OD2 | 0GA565@H6O | 95.90 (s) |
|  | Y227@OH | 4GA564@H6O | 33.39 (w) |
|  | D348@OD2 | 0GA565@H2O | 50.77 (m) |
|  | D348@OD1 | 0GA565@H4O | 25.39 (w) |
|  | Q399@OE1 | ROH563@HO1 | 75.91 (s) |
|  | Q399@OE1 | 4GA564@H6O | 19.04 (w) |
|  | 0GA565@O6 | R413@HH11 | 84.26 (s) |
|  | 0GA565@O6 | R413@HH22 | 19.04 (w) |
| Sucrose/MT complex | D81@OD1 | 1GA563@H4O | 99.55 (s) |
|  | D81@OD2 | 1GA563@H6O | 88.61 (s) |
|  | D81@OD1 | 1GA563@H6O | 15.64 (w) |
|  | D348@OD1 | 0CU564@H3O | 54.02 (m) |
|  | D348@OD2 | 1GA563@H2O | 24.24 (w) |
|  | D348@OD2 | 0CU564@H3O | 17.99 (w) |
|  | 1GA563@O3 | H347@HE2 | 37.78 (w) |
|  | 1GA563@O2 | H347@HE2 | 11.99 (w) |
| Maltose/MT complex | D81@OD2 | 0GA565@H6O | 98.20 (s) |
|  | D81@OD1 | 0GA565@H4O | 85.01 (s) |
|  | D81@OD2 | 0GA565@H4O | 25.64 (w) |
|  | D81@OD2 | Q191@HE22 | 15.49 (w) |
|  | D348@OD2 | 0GA565@H2O | 98.00 (s) |
|  | D348@OD1 | 0GA565@H3O | 96.00 (s) |
|  | 4GA564@O2 | H227@HE2 | 82.23 (s) |
|  | 4GA564@O2 | H227@HD1 | 17.44 (w) |
|  | 0GA565@O4 | R413@HH12 | 82.36 (s) |
|  | 0GA565@O6 | H124@HE2 | 13.39 (w) |

*Only hydrogen bonds with the occupations of more than 10% are shown: w = weak hydrogen bond, m = medium hydrogen bond, and s = strong hydrogen bond.
