# Supplementary material for: Insight into the substrate specificity change caused by the Y227H mutation of α-glucosidase III from the European honeybee (Apis mellifera) through molecular dynamics simulations
Source: PLoS One. 2018 Jun 4;13(6):e0198484. doi: 10.1371/journal.pone.0198484 (PMC5986129; doi:10.1371/journal.pone.0198484)
Supplement: S17 Table — (DOCX) [file pone.0198484.s028.docx]

**S17 Table.** Hydrogen bond occupations of third independent run of sucrose/WT, maltose/WT, sucrose/MT and maltose/MT complex from the 65 to 85 ns trajectory.

| System | DONOR | ACCEPTORH | Occupancy (%) |
| --- | --- | --- | --- |
|  | **res@atom** | **res@atom** |  |
| Sucrose/WT complex | D81@OD2 | 1GA563@H4O | 97.75 (s) |
|  | D223@OD2 | 1GA563@H6O | 91.25 (s) |
|  | D223@OD1 | 0CU564@H1O | 28.49 (w) |
|  | D348@OD2 | 1GA563@H3O | 62.42 (m) |
|  | D348@OD1 | 1GA563@H2O | 56.37 (m) |
|  | D348@OD2 | 1GA563@H2O | 41.58 (w) |
|  | D348@OD1 | 1GA563@H3O | 37.78 (w) |
|  | 1GA563@O4 | R413@HH11 | 77.01 (s) |
|  | 1GA563@O6 | H124@HE2 | 54.32 (m) |
|  | 0CU564@O4 | Y227@HH | 68.72 (m) |
| Maltose/WT complex | V167@O | ROH563@HO1 | 63.17 (m) |
|  | D223@OD2 | 0GA565@H4O | 73.86 (m) |
|  | Y227@OH | 4GA564@H6O | 13.89 (w) |
|  | D348@OD2 | 0GA565@H2O | 79.06 (s) |
|  | D348@OD2 | 4GA564@H3O | 76.51 (s) |
|  | D348@OD1 | 0GA565@H2O | 20.54 (w) |
|  | D348@OD1 | 4GA564@H3O | 19.29 (w) |
| Sucrose/MT complex | D81@OD2 | 1GA563@H6O | 95.20 (s) |
|  | D81@OD2 | 1GA563@H4O | 72.36 (m) |
|  | D81@OD1 | 1GA563@H4O | 48.83 (w) |
|  | D223@OD1 | 0CU564@H3O | 47.33 (w) |
|  | D223@OD2 | 0CU564@H3O | 46.88 (w) |
|  | D348@OD2 | 1GA563@H3O | 18.04 (w) |
|  | D348@OD2 | 1GA563@H2O | 54.37 (m) |
|  | D348@OD1 | 0CU564@H6O | 31.33 (w) |
|  | D348@OD1 | 1GA563@H2O | 27.69 (w) |
|  | 1GA563@O4 | R413@HH12 | 51.62 (m) |
|  | 1GA563@O3 | H347@HE2 | 20.84 (w) |
|  | 1GA563@O6 | H124@HE2 | 13.89 (w) |
| Maltose/MT complex | D81@OD2 | 0GA565@H6O | 95.55 (s) |
|  | D81@OD1 | 0GA565@H4O | 72.46 (m) |
|  | D81@OD2 | 0GA565@H4O | 50.92 (m) |
|  | D223@OD1 | 4GA564@H3O | 79.26 (s) |
|  | D223@OD2 | 0GA565@H2O | 89.36 (s) |
|  | D348@OD2 | 0GA565@H3O | 47.28 (w) |
|  | D348@OD1 | 0GA565@H3O | 12.19 (w) |
|  | Q399@OE1 | 4GA564@H6O | 13.29 (w) |
|  | 4GA564@O2 | H227@HE2 | 80.26 (s) |
|  | 0GA565@O6 | H124@HE2 | 79.36 (s) |
|  | 4GA564@O6 | K254@HZ2 | 11.89 (w) |
|  | 4GA564@O6 | K254@HZ3 | 12.64 (w) |
|  | 4GA564@O6 | K254@HZ1 | 13.74 (w) |
|  | 0GA565@O4 | R413@HH12 | 57.17 (m) |

*Only hydrogen bonds with the occupations of more than 10% are shown: w = weak hydrogen bond, m = medium hydrogen bond, and s = strong hydrogen bond.
